# Transmission of Severe Acute Respiratory Syndrome Coronavirus 2 to Close Contacts, China, January–February 2020

**DOI:** 10.3201/eid2709.202035

**Published:** 2021-09

**Authors:** Yu Li, Jianhua Liu, Zhongcheng Yang, Jianxing Yu, Chengzhong Xu, Aiqin Zhu, Hao Zhang, Xiaokun Yang, Xin Zhao, Minrui Ren, Zhili Li, Jinzhao Cui, Hongting Zhao, Xiang Ren, Chengxi Sun, Ying Cheng, Qiulan Chen, Zhaorui Chang, Junling Sun, Lance E. Rodewald, Liping Wang, Luzhao Feng, George F. Gao, Zijian Feng, Zhongjie Li

**Affiliations:** Chinese Center for Disease Control and Prevention, Beijing, China (Y. Li, J. Yu, A. Zhu, X. Yang, M. Ren, Zhili Li, J. Cui, H. Zhao, X. Ren, C. Sun, Y. Cheng, Q. Chen, Z. Chang, J. Sun, L.E. Rodewald, L. Wang, L. Feng, G.F. Gao, Z. Feng, Zhongjie Li);; Yichang Center for Disease Control and Prevention, Yichang, China (J. Liu, Z. Yang, C. Xu, H. Zhang, X. Zhou)

## Abstract

We estimated the symptomatic, PCR-confirmed secondary attack rate (SAR) for 2,382 close contacts of 476 symptomatic persons with coronavirus disease in Yichang, Hubei Province, China, identified during January 23–February 25, 2020. The SAR among all close contacts was 6.5%; among close contacts who lived with an index case-patient, the SAR was 10.8%; among close-contact spouses of index case-patients, the SAR was 15.9%. The SAR varied by close contact age, from 3.0% for those <18 years of age to 12.5% for those >60 years of age. Multilevel logistic regression showed that factors significantly associated with increased SAR were living together, being a spouse, and being >60 years of age. Multilevel regression did not support SAR differing significantly by whether the most recent contact occurred before or after the index case-patient’s onset of illness (p = 0.66). The relatively high SAR for coronavirus disease suggests relatively high virus transmissibility.

Transmissibility of an emerging infectious disease is a key factor for determining transmission dynamics in a population. The basic reproductive number, R_0_, indicates the average number of new cases resulting from 1 infected person in a completely susceptible population (*1*). In December 2019, an outbreak of coronavirus disease (COVID-19), caused by severe acute respiratory syndrome coronavirus 2 (SARS-CoV-2), was identified in Wuhan, Hubei Province, China (*2*). The mean R_0_ of COVID-19 was estimated to be in the range of 1.90–6.49 (*3*), indicating a high contagiousness that led to its rapid spread across the world (*4*). Another indicator of infectiousness is secondary attack rate (SAR), which is the probability that infection occurs among susceptible persons within a reasonable incubation period after known contact with an infectious person or an infectious source (*5*,*6*). Few estimates are available for the SAR for COVID-19 and its variation by type of contact, characteristics of index case-patients and contacts, and other factors. Information about factors associated with variation in SAR could help identify persons at high risk of transmitting the virus or acquiring COVID-19. Studies have reported transmission during the incubation period of COVID-19 (*7**–**10*) but with unclear quantification of risk. We estimated the SAR for COVID-19 and factors associated with risk for transmission.

## Methods

We conducted this study from January 23 through February 25, 2020, in Yichang, Hubei Province, China; the city has a population of ≈4 million. In accordance with National Health Commission guidelines for prevention and control of COVID-19 (http://www.gov.cn/xinwen/2020-01/23/content_5471768.htm), close contacts of COVID-19 case-patients were placed under 14-day quarantine for medical observation, during which time they would be tested by PCR for SARS-CoV-2 one time if illness symptoms developed but not tested if illness symptoms did not develop during the quarantine period.

Nasopharyngeal and pharyngeal swab samples from symptomatic quarantined persons were obtained and placed in airtight, freeze-tolerant tubes containing 3.5 mL of UTM (universal transport medium) viral transport medium. Sealed tubes were transported to the Yichang Center for Disease Control and Prevention laboratory (Yichang, China) within 24 hours of specimen collection. Viral RNA was extracted from samples and tested by using a commercial SARS-CoV-2 PCR diagnostic kit (Bioperfectus Technologies, https://www.bioperfectus.com) according to the manufacturer’s instructions. The commercial kit targets the open reading frame 1ab and nucleocapsid protein genes of the SARS-CoV-2 genome.

An index case-patient was defined as a person in this study with a positive SARS-CoV-2 PCR result. A close contact was defined as someone who had contact with an index case-patient without effective protection and within 1 meter, regardless of contact duration. Persons who had close contact with the index case-patient during or 2 days before the index case-patient’s illness onset were counted as close contacts. Secondary case-patients were close contacts with positive SARS-CoV-2 test results.

The types of contacts were considered mutually exclusive and were living together in the same household as an index case-patient, eating together (having meals together at a party, in a restaurant, or in another setting), caring for a patient (including doctors, nurses, and family members taking care of patients), sharing a vehicle (riding the same vehicle with an index case-patient but with no other close contact), or staying in a confined space (in the same confined space with an index case-patient, excluding in a vehicle, and with no other close contact). We included in our analyses close contacts who had completed their 14-day quarantine or who had positive SARS-CoV-2 results during quarantine in our study period. We excluded from our analyses close contacts of suspected case-patients for whom laboratory evidence of COVID-19 was lacking. We also excluded close contacts of >1 index case-patient or those whose information about contact type was missing.

We estimated the SAR by dividing the number of secondary cases by the number of close contacts. SAR in our study refers to secondary case-patients who had symptomatic, PCR-confirmed infection. We estimated the SAR for each type of close contact, tested statistical significance of differences by using χ^2^ or Fisher exact tests as appropriate, and considered p< 0.05 to be significant. We further analyzed factors significantly associated with SAR in univariate analyses with multilevel logistic regression mixed-effect models. We estimated crude and adjusted odds ratios (ORs) and 95% CIs, accounting for random effects of index case-patients.

Surveillance and analysis of close contacts of COVID-19 case-patients is part of public health surveillance in China. These procedures are exempted from need for institutional review board approval.

## Results

We included in our analyses 2,382 close contacts of 476 symptomatic index case-patients, all of whom completed their 14-day quarantine with assessed outcomes and who provided contact-related information (Figure). Close contacts were generally younger and more likely to be female than their corresponding index case-patients (Table 1). The overall SAR was 6.5%. SAR was 10.8% among close contacts who lived together with an index case-patient; this rate was significantly higher than that for other contact types, for which SAR ranged from 1.5% to 4.0% (Table 2). The SAR was 15.9% among spouses of index case-patients. SAR did not differ by sex of close contacts or of index case-patients. SAR increased with age, from 3.0% among close contacts <18 years of age to 12.5% among close contacts >60 years of age. A similar pattern by age was found for index case-patients (Table 2).

**Figure:**
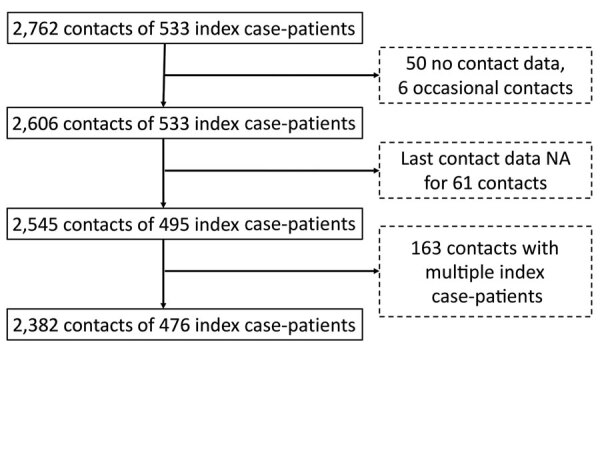
Enrollment of close contacts in study of transmission of severe acute respiratory syndrome coronavirus 2 to close contacts, China, January–February 2020.

**Table 1:** Characteristics of 476 index case-patients and 2,382 close contacts in study of transmission of severe acute respiratory syndrome coronavirus 2 to close contacts, China, January–February 2020*

Characteristic	Index case-patients	Close contacts
Age, y, mean (range)	49 (2–91)	43 (0–94)
Age group, y		
<18	5 (1)	267 (12)
18–59	339 (71)	1,559 (68)
>60	132 (28)	465 (20)
Sex		
M	262 (55)	1,162 (49)
F	214 (45)	1,198 (51)

*Values are no. (%) unless otherwise indicated.

**Table 2:** Secondary attack rate for coronavirus disease, overall and by characteristic, China, January–February 2020

Characteristic	Close contacts, no.	Secondary cases, no. (%)	p value
Overall	2,382	156 (6.5)	
Contact			
Type of contact			<0.001
Living together	1,020	110 (10.8)	
Eating together	835	33 (4.0)	
Care	80	2 (2.5)	
Sharing vehicle	68	1 (1.5)	
Stay in a confined space	379	10 (2.6)	
Most recent contact with index case-patient			0.023
Before illness onset	686	32 (4.7)	
After illness onset	1,696	124 (7.3)	
Whether contacts and index case-patients were spouses			<0.001
No	2,105	112 (5.3)	
Yes	277	44 (15.9)	
Close contacts			
Age			<0.001
<18 y	267	8 (3.0)	
18–59 y	1,559	89 (5.7)	
>60 y	465	58 (12.5)	
Sex			0.644
M	1,162	71 (6.1)	
F	1,198	83 (6.9)	
Index case-patients			
Age			<0.001
<18 y	86	0	
18–59 y	1,747	90 (5.2)	
>60 y	549	66 (12.0)	
Sex			0.704
M	1,303	82 (6.3)	
F	1,079	74 (6.9)	

The SAR was 4.7% for close contacts whose most recent contact with an index case-patient was during the index case-patient’s incubation period, compared with a SAR of 7.3% for close contacts for whom the most recent contact occurred after index case-patient illness onset (p = 0.023). In multilevel univariate analysis that accounted for index case-patient variation, the pattern of ORs for factors associated with SAR was similar to the pattern described above (Table 3). In multilevel analysis that used a multivariate model with age of close contact, adjusted ORs for the following differed slightly from those for the univariate analysis: age of index case-patient, type of contact, whether the close contact and the index case-patient were spouses, and most recent contact time between close contact and index case-patient. Associations between SAR and the most recent contact time with the index case-patient (before/after illness onset) and age of the index case-patients (<60 years/>60 years) were no longer statistically significant, although the directions of the associations were the same (Table 3). The associations of SAR with age of contact, living together with an index case-patient, and being the spouse of an index case-patient were still significant, although the point estimates of the adjusted ORs became smaller (Table 3).

**Table 3:** Univariate and multivariate analyses of factors associated with secondary attack rate for coronavirus disease, China, January–February 2020*

Characteristic of contact	Univariate			Multivariate†	
	Crude OR (95% CI)	p value		Adjusted OR (95% CI)	p value
Type of contact					
Not living together	Referent			Referent	
Living together	7.85 (3.89–15.83)	<0.01		5.12 (2.11–12.45)	<0.01
Spouse of index case-patient					
No	Referent			Referent	
Yes	6.46 (3.30–12.61)	<0.01		2.83 (1.31–6.11)	<0.01
Age of contact, y					
<60	Referent			Referent	
≥60	3.29 (1.86–5.82)	<0.01		2.61 (1.43–4.78)	0.01
Age of index case-patient, y					
<60	Referent			Referent	
≥60	5.13 (1.66–15.86)	<0.01		2.92 (0.80–10.59)	0.1
Most recent contact with index case-patient					
Before illness	Referent			Referent	
After illness	2.20 (1.06–4.59)	0.03		1.23 (0.49–3.07)	0.66

*Multilevel logistic regression model of mixed effects accounted for random effects of index cases. OR, odds ratio. †Other covariates included in the model were contact type, whether the contact was a spouse of the index case-patient, age of contact, age of index case-patient, and most recent contact with index case-patient.

## Discussion

We found the SAR among all close contacts to be 6.5%. Because confirmed case-patients were centrally isolated and away from home, the SAR we measured may be lower than it would have been under conditions of home isolation. Factors independently associated with significantly higher risk of contracting COVID-19 were living in the same house as an index case-patient, being a spouse of an index case-patient, and being older. We found evidence of presymptomatic transmission, in which close contacts who only had contact with a COVID-19 case-patient during the incubation period subsequently had positive SARS-CoV-2 test results. The SAR among these close contacts was 4.7%, significantly lower than that for contacts whose most recent contact occurred after illness onset of the index case-patient.

We estimated the COVID-19 SAR in a household to be 10.8%, slightly higher than SAR estimates for seasonal influenza and pandemic influenza (H1N1) viruses in Hong Kong (*11*). Our results suggest that transmissibility of SARS-CoV-2 might be similar or slightly higher than that of influenza virus, which has a SAR of ≈10% in the household setting (*11*). This similarity is consistent with the finding that the R_0_ for COVID-19 is also similar to or slightly higher than that for influenza (*12*). In contrast, the SAR in households is estimated to be 2%–7% for Middle East respiratory syndrome (*13*) and 6.2% for severe acute respiratory syndrome (*14*), suggesting slightly weaker transmissibility compared with COVID-19.

Our findings corroborated transmissibility of SARS-CoV-2 during the COVID-19 incubation period. Viral shedding has been observed during the COVID-19 incubation period (*15*,*16*). Our results were consistent with those of another study that estimated that 40% of the transmission events in COVID-19 clusters were attributed to presymptomatic virus transmission in China (*17*). Our multivariate analysis did not find statistically significant differences in SAR before and after illness onset, which is consistent with a SAR study in southern China that found infectivity during the incubation period to not differ statistically from infectivity after illness onset. Although respiratory signs such as coughing and sneezing after illness onset increased the probability of virus transmission compared with during the incubation period (*18*–*20*), studies suggest that viral load peaks right before illness onset (*10*,*21*), highlighting the threat for presymptomatic SARS-CoV-2 transmission.

Risk of contracting COVID-19 was positively associated with intimacy between contacts and index case-patients. Living in the same household with index case-patients considerably increased risk for COVID-19. Being a spouse of an index case-patient independently increased the risk of contracting COVID-19, consistent with findings from another study (P. Cui et al., unpub. data, https://www.medrxiv.org/content/10.1101/2020.02.26.20028225v2). However, the SAR was relatively low among contacts who provided care to patients, implying that risk for infection can be reduced by using protective equipment and by protective behaviors.

Previous studies indicated that age was associated with risk for severe and fatal infection (*22*); however, few studies directly assessed the effect of age on risk of contracting COVID-19. Our study confirmed that senior persons are at high risk for contracting COVID-19, highlighting the need to pay special attention to facilities with numerous seniors, such as nursing homes. However, our findings also suggested that older age does not necessarily increase the risk of transmitting the virus; our multivariate analysis found that the association between older age of index case-patients and SAR was not statistically significant, a finding consistent with a study showing that viral loads did not differ significantly by age (*10*).

The first limitation of our study is that for surveillance of close contacts, laboratory testing was initiated only when the contacts showed symptoms of illness. Asymptomatic infections with SARS-CoV-2 occur; for example, 1 study estimated that 17.9% of persons infected with SARS-CoV-2 did not have any symptoms (*23*). Therefore, our study will have missed asymptomatic case-patients and therefore underestimated the true SAR. Our estimates should therefore be interpreted as SAR limited to secondary case-patients with symptomatic COVID-19. The second limitation is that SAR is determined not only by infectiousness of the virus but also by protection levels, which might differ by geography, phase of the pandemic, education level of persons at risk, perceived threat from COVID-19, and other confounding factors. The third limitation is that the number of index case-patients <18 years of age and corresponding contacts was small; thus, our estimates of SAR for COVID-19 are more representative of transmissibility among adults than among children.

In conclusion, the SAR for COVID-19 is relatively high, suggesting relatively high transmissibility. This SAR is influenced by type of contact, level of intimacy between case-patients and contact, and age of contact. Our results provide additional evidence that SARS-CoV-2 can be transmitted by presymptomatic persons.
